# Effects of Estrogens on Adipokines and Glucose Homeostasis in Female Aromatase Knockout Mice

**DOI:** 10.1371/journal.pone.0136143

**Published:** 2015-08-28

**Authors:** Michelle L. Van Sinderen, Gregory R. Steinberg, Sebastian B. Jørgensen, Jane Honeyman, Jenny D. Chow, Kerrie A. Herridge, Amy L. Winship, Evdokia Dimitriadis, Margaret E. E. Jones, Evan R. Simpson, Wah Chin Boon

**Affiliations:** 1 MIMR-PHI Institute of Medical Research, Clayton Vic 3180 Australia; 2 The Florey Institute of Neuroscience and Mental Health, University of Melbourne, Parkville Vic 3000, Australia; 3 Dept of Anatomy and Developmental Biology, Monash University, Clayton Vic 3800, Australia; 4 St Vincent’s Institute of Medical Research and Dept of Medicine, University of Melbourne, Fitzroy, Vic 3065, Australia; University of Minnesota - Twin Cities, UNITED STATES

## Abstract

The maintenance of glucose homeostasis within the body is crucial for constant and precise performance of energy balance and is sustained by a number of peripheral organs. Estrogens are known to play a role in the maintenance of glucose homeostasis. Aromatase knockout (ArKO) mice are estrogen-deficient and display symptoms of dysregulated glucose metabolism. We aim to investigate the effects of estrogen ablation and exogenous estrogen administration on glucose homeostasis regulation. Six month-old female wildtype, ArKO, and 17β-estradiol (E2) treated ArKO mice were subjected to whole body tolerance tests, serum examination of estrogen, glucose and insulin, ex-vivo muscle glucose uptake, and insulin signaling pathway analyses. Female ArKO mice display increased body weight, gonadal (omental) adiposity, hyperinsulinemia, and liver triglycerides, which were ameliorated upon estrogen treatment. Tolerance tests revealed that estrogen-deficient ArKO mice were pyruvate intolerant hence reflecting dysregulated hepatic gluconeogenesis. Analyses of skeletal muscle, liver, and adipose tissues supported a hepatic-based glucose dysregulation, with a down-regulation of Akt phosphorylation (a key insulin signaling pathway molecule) in the ArKO liver, which was improved with E2 treatment. Concurrently, estrogen treatment lowered ArKO serum leptin and adiponectin levels and increased inflammatory adipokines such as tumour necrosis factor alpha (TNFα) and interleukin 6 (IL6). Furthermore, estrogen deficiency resulted in the infiltration of CD45 macrophages into gonadal adipose tissues, which cannot be reversed by E2 treatment. This study describes the effects of estrogens on glucose homeostasis in female ArKO mice and highlights a primary phenotype of hepatic glucose dysregulation and a parallel estrogen modified adipokine profile.

## Introduction

Glucose homeostasis is maintained throughout the body via essential cross talk between peripheral tissues. Elevated blood glucose levels following energy intake stimulate the release of insulin from the pancreas which in-turn, stimulates glucose uptake into peripheral organs such as skeletal muscle and adipose tissue. Additionally, the liver can produce glucose by glycogenolysis (i.e. the breaking down of glycogen) and by gluconeogenesis (i.e. the *de novo* synthesis of glucose from non-carbohydrate precursors such as lactate, pyruvate, glycerol and alanine). The former occurs more rapidly, beginning within two to three hours after a meal in humans, but the latter assumes a much greater importance with prolonged fasting [1,2]. Gluconeogenesis can also be stimulated by glucagon or inhibited by insulin to maintain homeostasis [3]. Dysregulated glucose homeostasis and obesity are key components of the metabolic syndrome (MetS). Excess adipose tissue produces and secretes multiple adipokines including leptin, adiponectin, resistin, visfatin; and in addition pro-inflammatory peptides, tumour necrosis factor alpha (TNFα), interleukin (IL) 6 and monocyte chemoattractant protein (MCP) 1, which are also released by invading macrophages commonly associated with obesity [4,5]. Adipokines and free fatty acids (FFA) released from adipocytes can also play a role in maintaining glucose homeostasis [3,6,7].

Both estrogens and androgens have been implicated in the modulation of various adipokines. This can be observed when investigating clinical changes involved with menopause and polycystic ovarian syndrome (PCOS). Postmenopausal women have low estrogens and this is associated with dyslipidemia, increased central adiposity, serum leptin and inflammatory markers with a decline in adiponectin [8]. The main source of estrogens after ovary senescence in postmenopausal women is from aromatase conversion of androgens in peripheral tissues such as adipose tissue and bone. Conversely, women afflicted with PCOS endure a state of hyperandrogenism which results in increased adiposity, leptin, inflammatory markers TNFα and IL6, and obesity independent decreases in adiponectin [9,10].

To elucidate the consequence of estrogen deficiency on glucose homeostasis and adipokine profiles, we utilized the aromatase knockout (ArKO) mouse model, which was generated via the deletion of exon 9 of the *Cyp19A1* gene. Aromatase is the rate-limiting enzyme for converting C19 androgens to C18 estrogens and therefore the ArKO mouse is unable to produce estrogens; yet it is still responsive to estrogens through active estrogen receptors (ER). This model is important because other models of estrogen deficiency, such as ovariectomy, retain the ability to produce extra-gonadal estrogen due to aromatization at extra-gonadal sites. This makes the ArKO a key model for investigating the effect of exogenous estrogen administration without uncontrolled endogenous interference.

## Materials and Methods

All efforts were made to minimize animal suffering and procedures were approved by the Monash Medical Centre Animal Ethics Committee (Permit Number: MMCB2008/08).

### Mice

Aromatase Knockout (ArKO) mice (C57Black6 X J129) were generated by disruption of the *Cyp19A1* gene [11]. Homozygous null or wild-type (WT) offspring were bred by crossing heterozygous ArKO mice and were genotyped by PCR [12]. Mice were housed in groups under pathogenic free conditions, fed a soy-free mouse chow (Glen Forest Stock feeders, Perth, Australia) and water *ad libitum* as previously described [12]. Female WT and ArKO mice at 6 months of age were used in these studies (n = 7–20 as stated in figure legends).

### Estrogen and Sham Treatments

Each ArKO mouse was implanted with a 17β-estradiol pellet (E2; 0.15 mg in 60 days i.e. 2.5 μg/day, for 6-weeks; Innovative Research of America, Toledo, OH, USA). No differences in body mass, organ weights or glucose tolerance were detected between 6 month-old untreated ArKO and littermates implanted with a placebo pellet (saline 60 day slow release; Innovative Research of America, Toledo, OH, USA; see S1 Table and S1 Fig), hence untreated 6 month-old ArKO female mice were used in this study. After treatment, mice were killed using a lethal dose of anesthetic (100mg/ml Ketamine and 20mg/ml Xylazine in PBS). Blood was collected by cardiac puncture and serum was separated, and stored at –20°C. Adipose, liver and muscle tissues were removed, weighed and snap frozen in liquid nitrogen and stored at -80°C.

### Insulin Stimulated Tissue Collection

Mice were anaesthetized (6mg pentobarbital sodium/100g body weight; Sigma, St Louis USA), and injected with insulin (150 U/kg; Actrapid; Novo Nordisk, Bagsvaerd, Denmark) after fasting overnight. Following a 5 min incubation, skeletal muscles (gastrocnemius, extensor digitorum longus, tibialis anterior and soleus), liver and gonadal fat were excised and rapidly frozen in liquid nitrogen for storage at -80°C. Fasting blood samples were collected, serum separated and stored at -20°C.

### Serum Estrogen Assay

Levels of 17β-estradiol in serum samples were measured in duplicates in a single RIA assay using commercially available kits and protocols from Diagnostic Systems Laboratories (E2, DSL-4800 ultra-sensitive; Webster, TX, USA). The theoretical sensitivity of detection was 2.2 pg/ml and the antibody used in this immunoassay was highly specific for 17β-estradiol. The intra-assay coefficients of variation (CV) for E2 were ≤8.9%.

### Insulin Assay

Insulin was measured by ELISA (#EZRMI-13K, Linco Research, St. Charles, MO, USA) following manufacturer’s protocol. Briefly, a 96-well microtitre plate pre-coated with monoclonal mouse anti-rat insulin antibodies was washed thrice with kit wash buffer before incubation with 10μl of control, standards or sample serum, plus a biotinylated anti-insulin antibody at room temperature. Unbound material was washed away and horseradish peroxidase was added to wells. Free enzyme conjugates were washed away and a light sensitive 3,3’,5,5’tetramethylbenzidine substrate added. Enzyme activity was measured spectrophotometrically at 450nm on the Envision Plate reader v 1.09 (Perkin Elmer, Waltham, MA, USA).

### Tolerance Tests

All mouse cohorts were subjected to glucose tolerance test (GTT– 1g glucose/kg of body weight i.p, after 8 h of fasting; Sigma, St Louis USA), insulin tolerance test (ITT– 0.5U insulin/kg of body weight i.p., after 8 h of fasting; Actrapid; Novo Nordisk, Bagsvaerd, Denmark) and pyruvate tolerance test (PTT– 1g pyruvate/kg of body weight i.p., after ~16-20h of fasting; Sigma, St Louis USA). At least five days of recovery were allowed between each test. Tail bleeding at specific time point was used to obtain blood samples which were analyzed for glucose content (AccuChek Performer, Roche, Mannheim, Germany) immediately before, and at 20, 40, 60, 90 and 120 min after an intraperitoneal injection.

### Ex-vivo Muscle 2-deoxyglucose (2-DG) Uptake Assay

Soleus and extensor digitorum longus (EDL) muscles from WT and ArKO mice were dissected from anesthetized 6 month-old female mice and *ex vivo* 2-DG uptake was measured with or without insulin (2.8 nM Actrapid; Novo Nordisk, Bagsvaerd, Denmark) as previously described [13]. Muscle 2-DG uptake was measured by liquid scintillation counting (Tri-Carb 2000, Packard Instrument) of the muscle lysate/supernatant which was prepared by homogenization of the tissue sample in ice-cold protein lysis buffer (50mM HEPES, pH 7.4, 150mM NaCl, 10mM NaF, 1mM sodium pyrophosphate, 0.5mM EDTA, 250mM sucrose, 1mM DTT, 1mM Na_3_VO_4_, 1% Triton X-100 and 1 Roche protease inhibitor tablet per 50ml) followed by centrifugation at 4°C, 13,000 rpm for 30 min and collected.

### Western Analysis

Ice-cold protein lysis buffer (2.5mM HEPES, 68.5mM NaCl, 0.5mM MgCl_2_, 0.5mM CaCl_2_, 5mM NaF, 1mM EDTA, 5mM Na pyrophosphate, 1mM NaVO_4_, 0.5% Nonidet P40, and 5% glycerol) containing protease inhibitors (Complete Mini; Roche, Mannheim, Germany) was incubated with tissue samples for 45 min on ice as a form of homogenization. Lysate supernatants were collected after centrifugation at 14,000*g* for 15 min, and stored at -20°C. Protein content was determined by the bicinchoninic acid (BCA) method (Pierce Biotechnology, Rockford, IL, USA). Protein samples (50μg) added to 1× sample loading buffer (0.125M Tris-Cl, 4% SDS, 20% v/v Glycerol, 0.2M DTT, 0.02% Bromophenol Blue, pH 6.8), heated for 5 min at 95°C, separated by SDS-PAGE (10% polyacrylamide resolving gel and 4% stacking gel) and transferred to a nitrocellulose membrane (Amersham GE Healthcare, Piscataway USA). Primary antibodies diluted in 1% BSA in Tris Buffered Saline with 1% Tween were incubated rocking overnight at 4°C (mouse anti-Phospho-Akt (Ser473) 1:1000, rabbit anti-Akt 1:1000; Cell Signaling Technologies, Beverly MA, USA). Appropriate Alexa Fluor secondary antibodies were incubated at room temperature for 1 h. Protein band intensities were quantified using the Odyssey infrared imaging system (Licor Biosciences, Lincoln, NE, USA). All protein quantifications were normalized to housekeeping protein β-tubulin (mouse anti-β-tubulin; 1:10,000, Millipore, California USA).

### Real-time PCR

Total RNA was extracted from mouse gonadal adipose and liver tissues using RNeasy RNA extraction kit (QIAGEN, Duesseldorf, Germany) and Ultraspec RNA isolation system (Fisher Biotec, WA, Australia) following manufacturers’ protocols, respectively. All RNA samples were DNase I treated (DNAfree, Ambion, Foster City, CA, USA). Total RNA concentrations were quantified spectrophotometrically using the NanoDrop1000 (Thermo, Willmington, USA). cDNA derived from reverse transcription (Roche, Mannheim, Germany) of 1μg of RNA was diluted 10-fold and amplified by real-time PCR in the ABI7900H (Applied Biosystems, California) using a SYBR ‘Master Mix’ (DMSO (Sigma) 10X Gold PCR Buffer, AmpliTaqGold (Applied Biosystems), 1M Mg(oAC)_2_ (Sigma), 100mM dACT, dCCT, dGCT and dTCT (Bioline) 1μg/μl 6-ROX and 10,000X con SYBR (Invitrogen)0, with specific oligonucleotide pairs:

Adiponectin (**F:** ^5’^TGTTGGAATGACAGGAGCTGA^3’^, **R:** ^5’^CACACTGAAGCCTGAGCGATAC^3’^); Interleukin-6 (IL6) (**F:** ^5’^ATGGATGCTACCAAACTGGAT^3’^, **R:** ^5’^TGAAGGACTCTGGCTTTGTCT^3’^); Leptin (**F:** ^5’^TCCAGAAAGTCCAGGATGACAC^3’^, **R:** ^5’^CACATTTTGGGAAGGCAGG^3’^);

Monocyte chemoattractant protein 1 (MCP-1; also referred to as chemokine (C-C motif) ligand 2, CCL2) (**F:** ^5’^CCACTCACCTGCTGCTACTCA^3’^, **R:** ^5’^TGGTGATCCTCTTGTAGCTCTCC^3’^); tumour necrosis factor α (TNFα) (**F:** ^5’^CTCTTCAAGGGACAAGGCTG^3’^, **R:** ^5’^GGACTCCGCAAAGTCTAAG^3’^). Real-time PCR data were calculated using ∆∆CT method of analysis. All samples were normalized to housekeeping gene cyclophilin (**F:** ^5’^CTTGGGCCGCGTCTCCTTC^3’^, **R:** ^5’^TGCCGCCAGTGCCATTAT^3’^) transcript levels.

### Adipokine Level Assays

Adipokine levels in mouse serum samples were analysed using the adipokine LINCOplex Kit 96 well assay according to manufacturer’s protocol (Mouse Serum single-plex adiponectin panel #MADPK-71-ADPN; Mouse Serum Adipokine kit #MADPK-71K –leptin, MCP1, TNFα, IL-6, PAI and insulin; Millipore, California USA). Thawed serum samples were vortexed and centrifuged before use. Mouse adipokine standards and quality control cocktails provided were reconstituted in deionized water. Briefly, standards, quality controls and serum samples were pipetted into individual wells of the 96-well microtitre filter assay plate in duplicates after assay plates had been washed in a 1× wash buffer with a vacuum manifold filtration unit (Millipore, California USA). After 4°C overnight incubation with antibody immobilized beads, plates were washed before mouse adipokine detection antibodies were added, and incubated for 30 min. Unhybridized antibodies were washed off before Streptavidin-Phycoerythrine were added and incubated at room temperature for 30 min. After Luminex sheath fluid was added, plates were scanned and analysed on the Luminex 100 instrument and software (Luminex Corporation, Texas, USA).

### Liver Tissue Triglyceride (TG) Content

Saponified extracts were prepared from frozen liver samples, and the triglyceride content was quantified by comparison to a glycerol standard curve as previously described [14]. Hepatic triglycerides (TG) were extracted by digesting 100–300 mg liver tissues overnight at 55°C in ethanolic potassium hydroxide (2:1 vol/vol of 100% ethanol and 30% potassium hydroxide) [15]. Samples were neutralized with water:ethanol (1:1 vol/vol). After centrifugation (8,000×g, 5 min), supernatant was collected and further diluted to 1.2ml with water:ethanol, of which 200μl proceeded to saponification with 215μl 1M MgCl_2_ by vortexing briefly and incubating at 4°C for 10 min. Saponified liver extracts (the upper phase) were separated by centrifugation, and the glycerol content was quantified by mixing 6μl extract with 200μl Free Glycerol Reagent (Sigma-Aldrich, Missouri, USA), which would react with free endogenous glycerol to generate a dye with an absorbance at 540nm. Absorbance (measured using the Wallac 1420 Victor Plate Reader, LabX, Midland ON Canada) is directly proportional to free glycerol concentration of the sample. All blanks (6μl water), glycerol standards (6μl diluted 1:3; Sigma-Aldrich, Missouri, USA) and samples were quantified in duplicates.

### Immunohistochemistry

Paraffin embedded gonadal adipose tissues were sectioned (5μm), dewaxed in histosol (Sigma-Aldrich) and rehydrated in a graded series of ethanol. Sections were microwaved at high power (700 W) in 0.01 M citric acid buffer (pH 6.0) for 5 min. Endogenous peroxidase activity was quenched with 6% H_2_O_2_ in 100% methanol (1:1 v/v) for 10 min. Tissues were incubated with non-immune blocking solution (10% normal horse serum, 2% normal mouse serum) diluted in 1×Tris-buffered saline for 30 min. The primary antibody; rabbit polyclonal CD45 (Abcam #ab10558) or non-immune goat IgG (isotype negative control, DakoCytomation) were applied at 1μg/ml in blocking solution for 18 h at 4°C, followed by biotinylated goat anti-rabbit IgG (Dako 1:200) for 30 min, then streptavidin–biotin–peroxidase complex ABC (DakoCytomation Glostrup, Denmark) according to the manufacturer's instructions. Peroxidase activity was visualized by application of diaminobenzidine substrate (DakoCtyomation) for 2 min. Tissues were counterstained with Harris haematoxylin (Sigma), air dried and mounted. A quality control slide was present in each immunohistochemistry run. Five representative photographs at 20× magnification were taken from each tissue section (n = 5 animals per group) using CellSense software (Olympus). The number of CD45 positive cells per field were counted and averaged for each tissue by an observer who was blind to the genotypes and treatment.

### Statistical Analysis

All statistical analyses were performed using GraphPad Prism version 5.04 for Windows (GraphPad Software Inc). Data were analyzed using a two-tailed t-test or Mann-Whitney test depending on the outcome of Shapiro-Wilk normality test. Since the data of body weight, liver triglyceride, serum glucose, adipocyte leptin and adiponectin, and ex-vivo muscle glucose uptake passed the Shapiro-Wilk normality test, they were analyzed by two-tailed t-test. Other data were analyzed by two-tailed Mann-Whitney test. Data are expressed as mean ± standard deviation (SD). Differences between groups are considered statistically significant if p-value <0.05.

## Results

### The effects of estrogens on body and tissue weights

Untreated female ArKO mice were significantly heavier than WT controls (Table 1), and, their gonadal (omental) adipose tissue accumulation was higher (p<0.05, Table 1) than WT. 17β-estradiol (E2) treatment (2.5μg/day) led to a reduction in body weight and gonadal adipose tissue weights compared to both untreated ArKO (p<0.05, p<0.01; Table 1) and WT littermates (p<0.05, p<0.01; Table 1). Untreated ArKO uteri were significantly reduced in wet weight (p<0.01) as compared to the WT counterparts. Upon E2 treatment, ArKO uterus weight surpassed that of WT size (p<0.0001 vs. ArKO). Female ArKO livers tended to be heavier than the WT livers (p = 0.053, Table 1) but no differences were seen in liver weights between ArKO+E2 and WT.

**Table 1:** Female WT, ArKO mouse body, tissue weights, serum 17β-estradiol levels and liver triglycerides levels.

	WT	KO	KOE
Number of mice	7–10	7–10	6–13
Body weight (g)	26.74±2.606	32.21±9.85*	27.14±3.78#
Gonadal/Omental adipose weight (g)	0.71±0.19	1.14±0.5*	0.36±0.35** ##
Uterus weight (g)	0.11±0.01	0.01±0.003**	0.27±0.25###
Liver weight (g)	1.04±0.18	1.47±0.76 (p = 0.053 vs WT)	1.21±0.13
Serum 17β-estradiol (pM)	13.02±4.51	Not detectable	136.7±209 (p = 0.051 vs WT)
Liver Triglycerides (mg/ml)	0.84±0.33	1.96±1.42*	0.63±0.22#

Body, gonadal adipose tissue, and uterus weights in grams (g) of 6 month-old female wild type (WT), Aromatase knockout (KO) and 2.5μg/day 17β-estradiol-treated ArKO (KOE). Serum estrogen levels were measured (pM) in 6 month-old female WT, KO and KOE animals. Liver triglycerides were measured (mg/ml) in 6 month old female WT, KO and KOE. Data are expressed as mean ± SD

* p<0.*05*, and

**p<0.*01* versus WT mice.

^#^ *p<0*.*05*

^##^ *p<0*.*01*

^###^ *p<0*.*001* versus ArKO.

### Serum estrogens levels

Serum estrogens in untreated ArKO mice are undetectable. After 6 weeks of E2 treatment, serum estrogen levels in the ArKO mice were increased compared to both the untreated ArKO and WT mice (p = 0.05; Table 1).

### Liver triglyceride levels

Mean liver triglyceride levels of untreated ArKO (1.96 mg/ml) are more than 2-fold higher (p<0.05) than that of WT (0.84 mg/ml), which were returned to WT levels upon E2 treatment (0.63 mg/ml; p<0.05 vs untreated ArKO; Table 1).

### Whole body glucose, pyruvate and insulin tolerance in the ArKO mice

**Fig 1:**
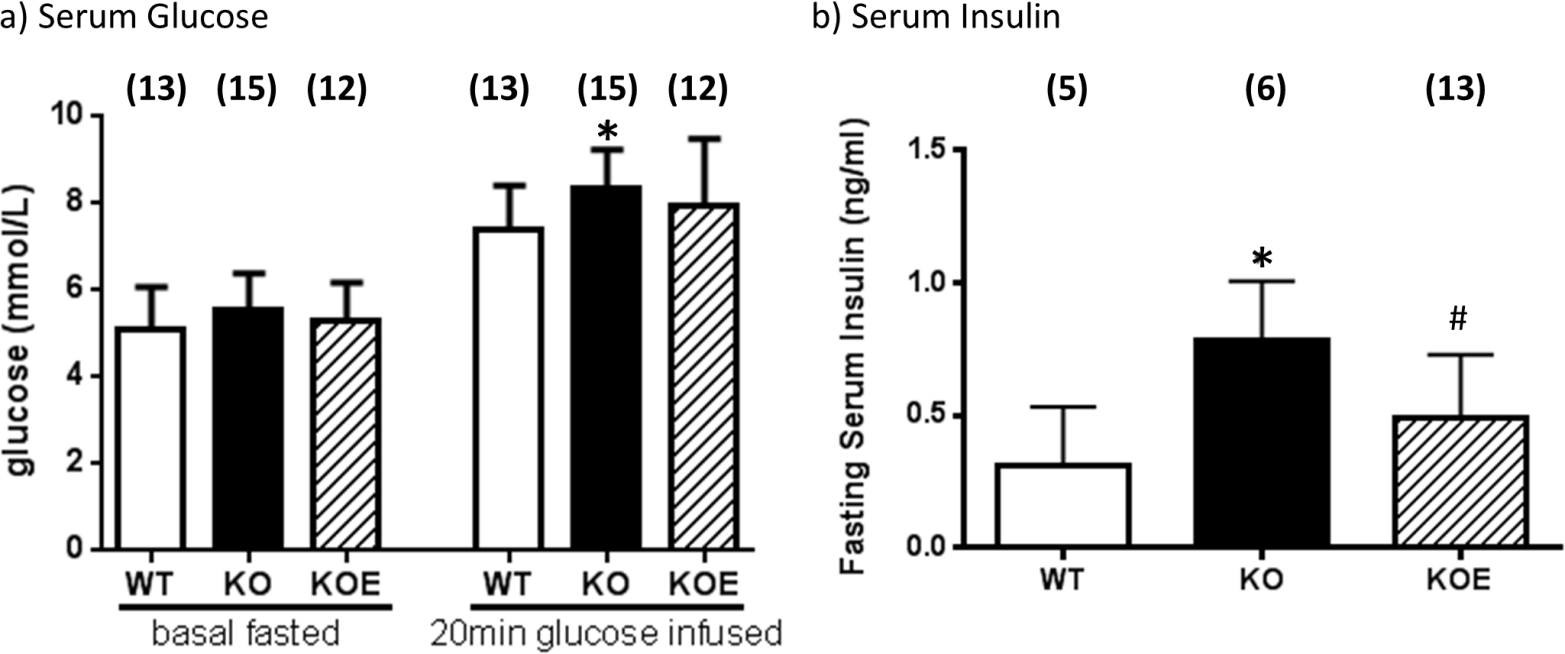
Serum glucose and insulin levels. **(a)** Fasted basal serum glucose and 20 min after glucose challenge serum glucose levels; **(b)** Fasted basal serum insulin (from 6 month-old female WT (wild type), KO (aromatase knockout) and KOE (aromatase knockout treated with 2.5µg/day 17β-estradiol) mice. Data are expressed as mean ± SD (n = 5–13 per group).

Basal fasted serum glucose levels were similar amongst the 3 groups of mice (Fig 1A). However, 20 min after glucose challenge, untreated ArKO mice serum glucose levels were significantly higher (p<0.05) than WT counterparts, and E2 treatment attenuated this increase (Fig 1A). Basal fasted serum insulin levels were increased in the ArKO mice (p<0.05) and was reduced upon E2 treatment (p<0.05; Fig 1B).

**Fig 2:**
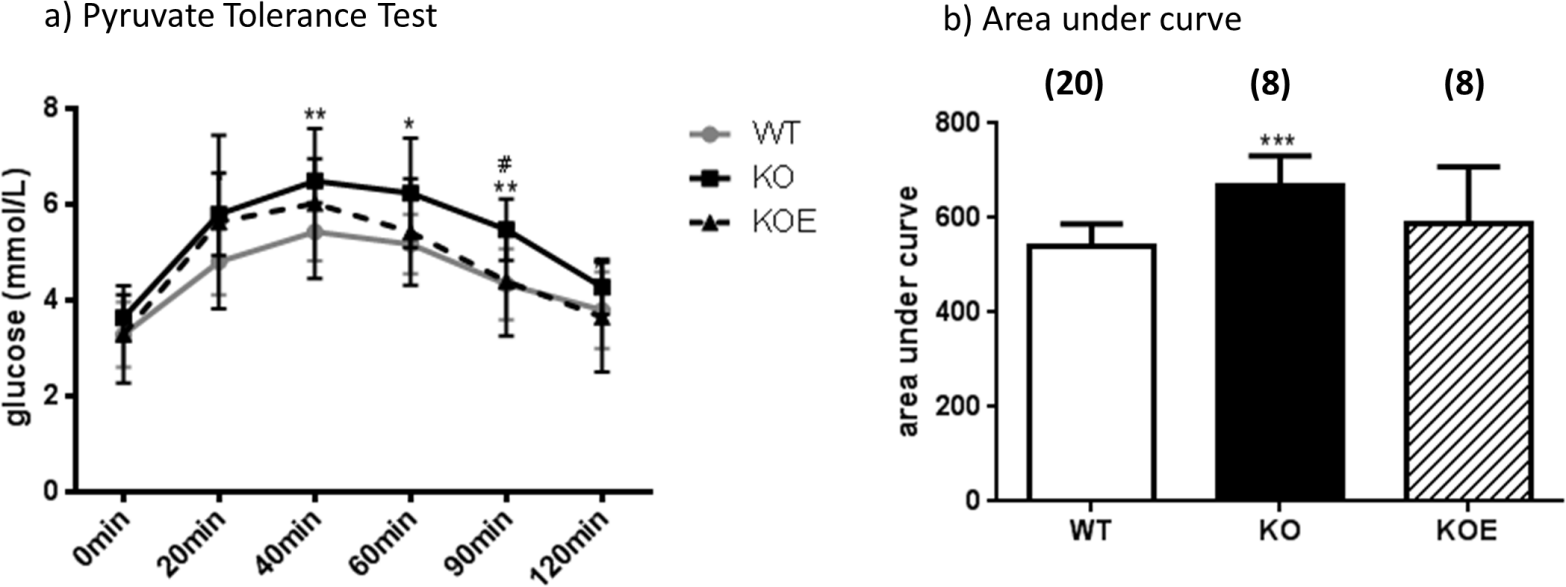
Whole body glucose, insulin and pyruvate tolerance. **(a)** Whole body tolerance tests were completed on fasted 6 month-old female wildtype (WT) and aromatase knockout (KO) and 2.5μg/day 17β-estradiol-treated KO (KOE) pyruvate tolerance test and **(b)** corresponding area under curve. Data are presented as the mean ± SD. (n = shown on corresponding bar/ group) *p<0.*05*, **p<0.*01*, and ***p<0.*001* versus expression in WT samples and ^*#*^ *p<0*.*05 and* ^*##*^ *p<0*.*01* versus KO samples.

No significant differences were seen in area under the curve (AUC) for whole body glucose and insulin tolerance tests between all 3 groups (data not shown). Untreated female ArKO mice displayed significantly higher pyruvate intolerance (p<0.001 AUC, Fig 2) compared to WT. Upon E2 treatment, the ArKO mice no longer show higher pyruvate intolerance as compared to WT, suggesting that the primary metabolic defect in ArKO mice is related to elevated hepatic gluconeogenesis.

### The role of estrogens and androgens in ex-vivo muscle glucose uptake

**Fig 3:**
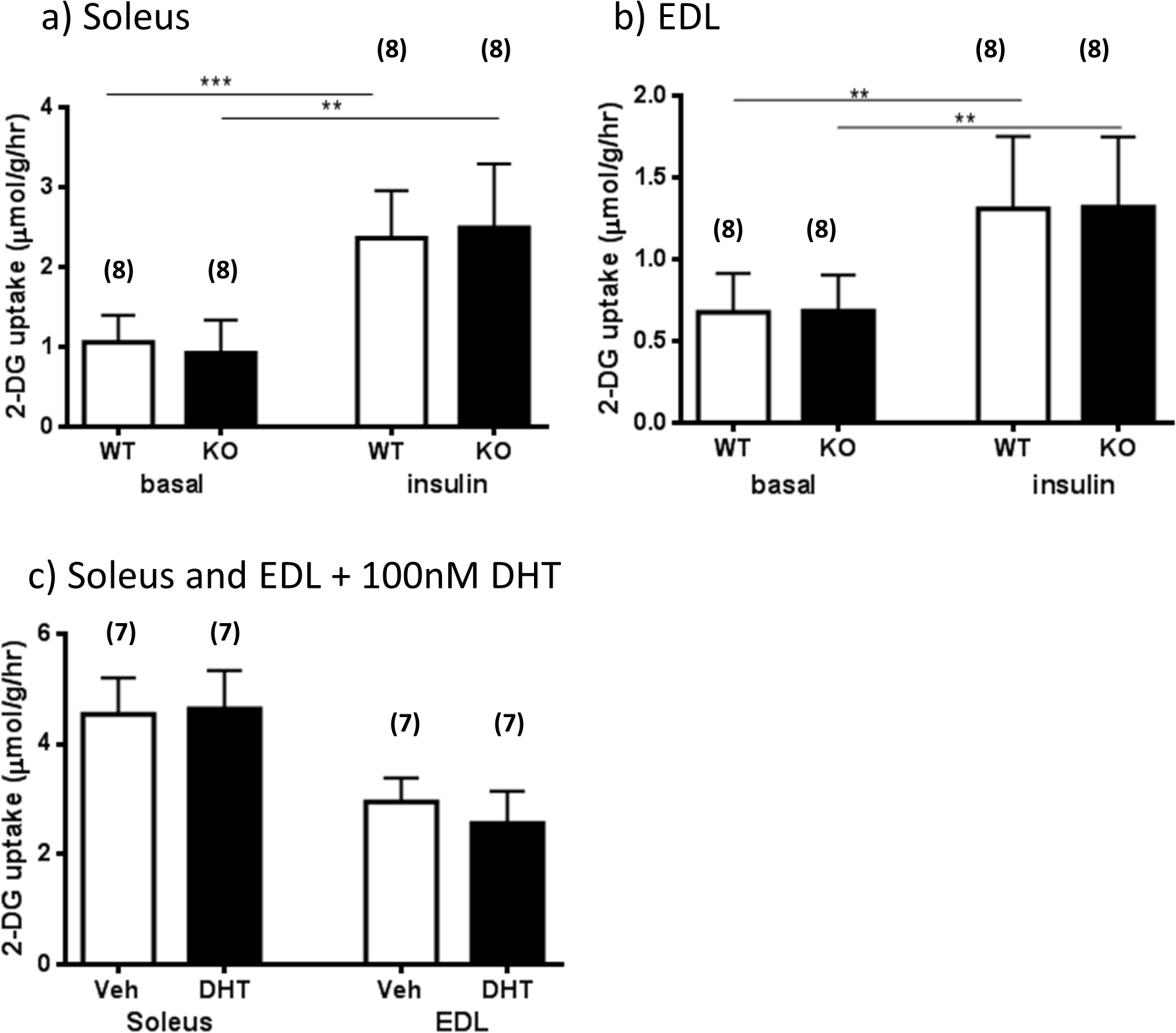
The role of estrogens and androgens in ex-vivo muscle 2-deoxy-glucose (2-DG) uptake. Six month-old wildtype (WT) and aromatase knockout (KO) female *ex vivo* 2-DG uptake in basal or insulin stimulated **(a)** soleus and **(b)** EDL muscle extractions. Data are presented as mean ± SD (n = 7/group). **p<0.01 and ***p<0.*001* expression in basal versus insulin stimulated muscle. Six month-old wildtype (WT) female *ex vivo* glucose uptake in **(c)** vehicle or dihydrotestosterone (DHT) (100nM) treated soleus and EDL muscle extractions. Data are presented as mean ± SD (n = 7/group).

Consistent with the idea that the liver was the primary site of metabolic dysfunction, muscles (EDL and soleus) from both untreated ArKO and WT mice showed similar efficacy in basal or insulin stimulated glucose uptake *ex vivo* (Fig 3A and 3B). The validity of this experiment was confirmed by significant increases of the glucose uptake observed in insulin stimulated muscles compared to vehicle treated muscles in both WT and KO genotypes (soleus, p<0.001 and 0.001 respectively; EDL, p<0.01 and 0.01 respectively). Since ArKO mice have higher serum androgen levels [16], we further examined the direct effects of dihydrotestosterone (DHT, 100nM) on the glucose-uptake of six month-old WT soleus and EDL muscles in the *ex vivo* experiment. No differences were found in soleus nor EDL insulin stimulated glucose uptake between vehicle and DHT treated muscles (Fig 3C, P>0.05) supporting the idea that androgens have little effect on muscle insulin sensitivity.

### Gonadal adipose tissue, liver and muscle Akt protein phosphorylation

**Fig 4:**
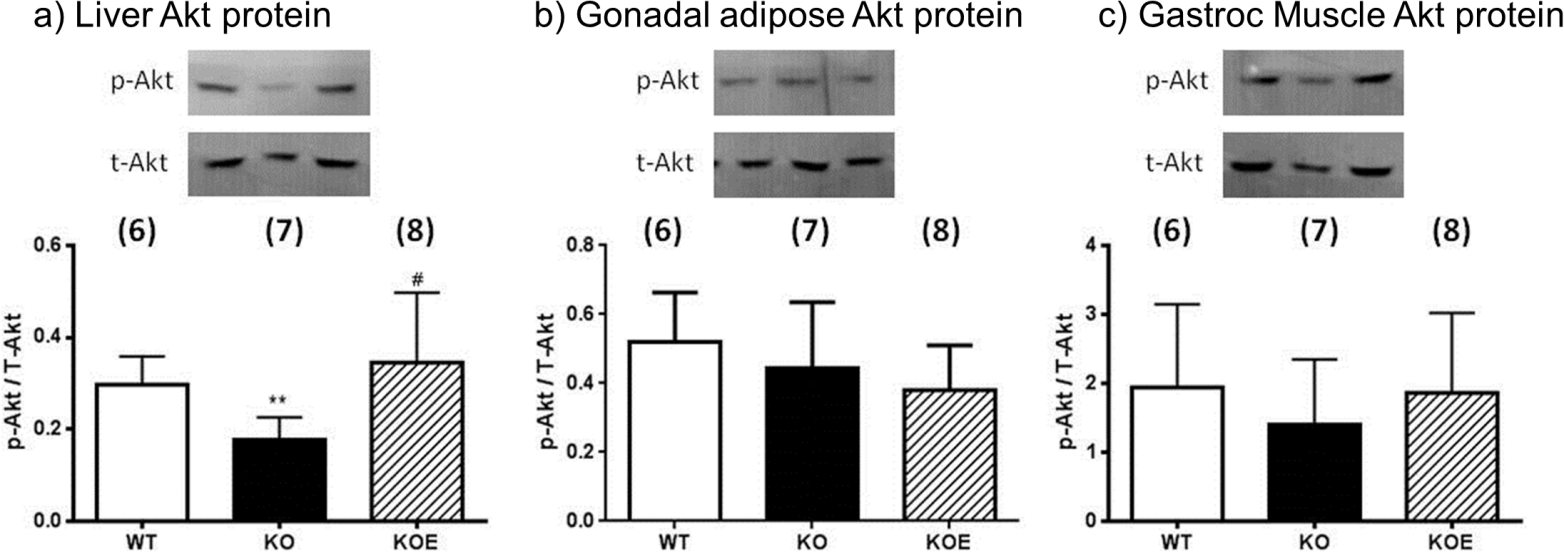
Insulin stimulated Akt protein phosphorylation in liver, adipose and skeletal tissues. Six month-old female wildtype (WT) and aromatase knockout (KO) and 2.5μg/day 17β-estradiol-replaced KO (KOE) mice were used. Protein phosphorylation analyses of Akt levels were performed on protein extracted from insulin stimulated (a) liver, (b) adipose tissue and (c) skeletal muscle. Data are presented as mean ± SD (n = 6-8/group). **p<0.*01* versus expression in age-matched *WT* samples and ^*#*^ *p<0*.*05* versus expression in age-matched ArKO untreated samples.

To determine the potential mechanisms responsible for differences in glucose homeostasis, the expression and phosphorylation levels of Akt (a critical component of canonical insulin signaling) was measured in gonadal adipose, skeletal muscle and liver tissues of six month-old WT and ArKO female mice after an i.p. injection of insulin. Consistent with our metabolic data indicating defects in liver glucose homeostasis, we found that liver Akt phosphorylation was significantly decreased (p<0.05) in the untreated ArKO mice compared to the WT and was restored with E2 administration (Fig 4A). There were no differences in Akt phosphorylation in adipose tissue or skeletal muscle (Fig 4B and 4C).

### Adipokine expression levels in female ArKO mice

**Fig 5:**
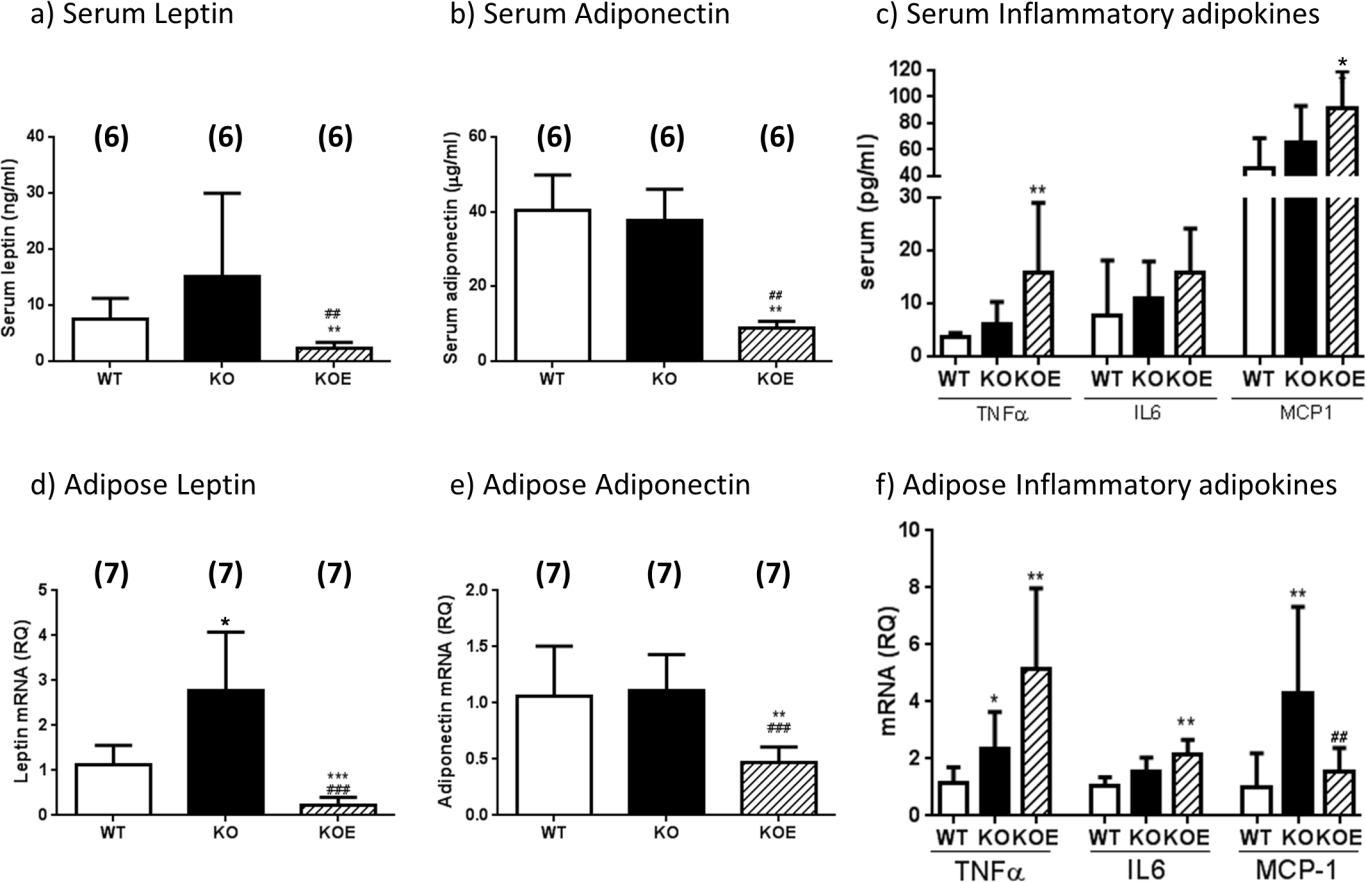
Serum adipokine and gonadal adipose tissue adipokine transcript levels. Serum adipokine analyses of **(a)** leptin, **(b)** Adiponectin and **(c)** TNFα, IL6 and MCP1 concentrations were performed on serum from six month-old female wildtype (WT), Aromatase knockout (KO) and 2.5μg/day 17β-estradiol-treated KO (KOE) mice. Data are presented as mean ± SD (n = 6/group). *p< 0.*05*, **p<0.*01*, versus expression in age-matched *WT* samples and #*#p<0*.*01* verses KO samples. Real-time-PCR analyses of **(d**) Leptin, **(e)** Adiponectin, **(f)** TNFα, IL6 and MCP1 gene expression were performed on cDNA derived from total RNA prepared from gonadal adipose tissue of six month-old female wildtype (WT), Aromatase knockout (KO) and 2.5μg/day 17β-estradiol-treated KO (KOE) mice. Data are presented as mean ± SD (n = 7/group). following normalization to cyclophilin *p<0.*05*, **p<0.*01*, ***p<0.*001* versus expression in age-matched WT samples and ^##^ *p<0*.*01 and* ^###^ *p<0*.*001* verses KO samples.

To examine the mechanisms potentially contributing to the development of liver insulin resistance in ArKO mice, we examined serum adipokines and markers of inflammation. Serum leptin levels in untreated ArKO mice tended to be increased as compared to WT, whilst leptin transcript levels were significantly increased in the gonadal adipose tissue (p<0.05; Fig 5A and 5D). After E2 treatment, ArKO serum leptin levels (p<0.01 and p<0.01) as well as transcript levels (p<0.001 and p<0.001) were lowered as compared to both ArKO and WT respectively (Fig 5A and 5D).

Both serum adiponectin and adipose adiponectin transcript levels were similar in the untreated ArKO and WT littermates (Fig 5B and 5E). Unexpectedly, E2-treatment significantly decreased serum adiponectin and adipose adiponectin mRNA levels compared to both WT and untreated ArKO mice (p<0.01 and p<0.01 respectively).

Levels of serum inflammatory adipokines TNFα, IL6 and MCP1 remained comparable between untreated female ArKO and their WT littermates (Fig 5C) although significant increases in adipose TNFα and MCP1 transcript levels (p<0.05 and p<0.01 respectively) were detected. Following E2 treatment, serum inflammatory markers TNFα and MCP1 showed significant increases (p<0.01 and p<0.05 respectively) compared to WT littermates. This same trend was observed for serum IL6 (p<0.1) when ArKO mice were treated with E2 (Fig 5C). While adipose TNFα and IL6 mRNA levels were increased following E2 treatment (p<0.01 and p<0.01) compared to WT, MCP1 mRNA levels remained comparable to WT levels (Fig 5F). No differences in serum Plasminogen Activation Inhibitor (PAI) were observed between all treatment groups (data not shown).

### Macrophages in gonadal/omental adipose tissue

**Fig 6:**
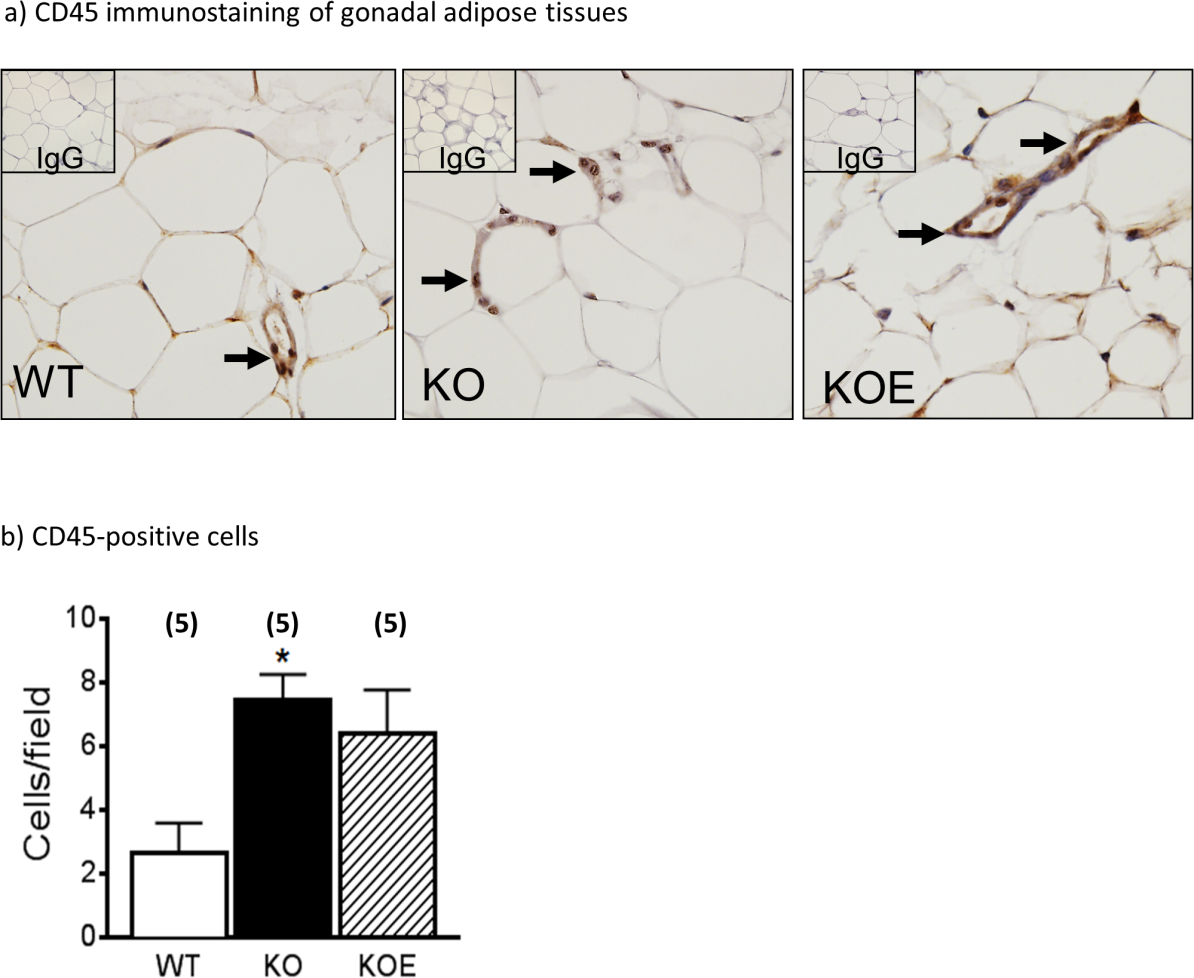
CD45 Immunohistochemistry of gonadal adipose tissues. **(a)** Representative CD45 immunohistochemistry staining of gonadal adipose tissues from six month-old female wildtype (WT), aromatase knockout (KO) and 2.5μg/day 17β-estradiol-treated KO (KOE) mice; **(b)** quantitated CD45-positive cells. Data are presented as mean ± SD (n = 5/group). *p< 0.*05*, versus age-matched WT samples.

A possible contributing source of inflammatory adipokines besides adipocytes is adipose associated macrophages which are CD45 positive [17]. Thus, we used CD45 immunostaining to examine the extent of macrophage invasion into the gonadal adipose tissue (Fig 6A). CD45 positive cells were significantly increased in untreated ArKO mice as compared to WT (p<0.05; Fig 6B). E2 treatment in the ArKO mice was unable to reduce CD45 positive cell population (p = 0.058 vs WT).

## Discussion

The role of estrogens in the regulation of glucose homeostasis has been extensively studied as recently reviewed by Kim et al. and Mauvais-Jarvis et al. [18,19]. However, defining the mechanisms by which exogenous estrogens affect glucose homeostasis, without the interference of endogenous production, has yet to be elucidated. This is important when considering the effects of hormone replacement therapy for menopause (when the ovaries, the main source of estrogens within the body, have ceased hormone production).

This is the first study to use whole body tolerance tests in female ArKO mice to measure whole-body glucose homeostasis. Female ArKO mice showed an increase in gonadal adipose tissue, serum glucose 20 min after glucose challenge, and fasted basal insulin levels compared to WT counterparts, indicating defects in glucose homeostasis. Pyruvate tolerance tests indicated that the primary defect in glucose control was attributed to elevated hepatic gluconeogenesis in female ArKO mice. In comparison, previous studies of the **male** ArKO mice showed similarly increased adiposity and serum glucose as well as glucose and pyruvate intolerance [20].

Higher liver triglycerides, which were reduced upon estrogen treatment, in the female ArKO mice also support recent finding that liver fat is the best predictor of liver insulin sensitivity [21]. Consistent with this finding, treatment of female ArKO mice with insulin revealed significant reduction in liver phosphorylation of Akt, the primary signal controlling hepatic gluconeogenesis. These effects appear to be independent of changes in systemic inflammation.

Although, no changes were seen in whole body glucose or insulin tolerance (AUC) between the female ArKO and WT mice, the former were found to be pyruvate intolerant. Upon further investigation of peripheral tissue involvement, we revealed a sensitivity of the liver to the presence of estrogens. Liver Akt phosphorylation in the ArKO mouse was decreased and improvements were noted upon E2 treatment. Akt is a key downstream mediator in the insulin signaling pathway and is able to mediate reductions in gluconeogenesis via PGC1α inhibition [22] and FOXO1 phosphorylation [23,24]. These factors combined suggest signs of hepatic insulin resistance in the ArKO mouse. This hepatic phenotype of glucose intolerance along with modifications to genes involved in gluconeogenesis, insulin signalling and lipid synthesis were also seen in the male ArKO mice as previously published [20].

Further analysis of the ArKO peripheral tissues also suggested that there may be a role for the adipose tissue in the dysregulation of glucose homeostasis seen in the female ArKO mice. In the adipose tissue, the production of adipokines revealed a negative correlation between increased leptin with decreased estrogen levels in the ArKO mice as noted previously [25]. Estrogens are a known stimulator of leptin expression as evident by fluctuations during the estrus cycle, and inhibition upon administration of the anti-estrogen drug tamoxifen [26–28]. Thus, the increased leptin expression in the ArKO mice and its prominent reduction upon estrogen treatment are more likely an effect of changes in excessive gonadal adiposity than a direct effect of estrogens. By contrast, adiponectin levels were reduced in the ArKO mice with E2 administration in accordance with previous *in vivo* [29,30] and *in vitro* [31] studies demonstrating the estrogen suppression of adiponectin. This suggests that estrogen action on adiponectin, which is an insulin sensitizer, may play an important role in glucose homeostasis. Choi et al. [32] described the role of PPARγ, causing direct upregulation of adiponectin. On this note, we have previously reported that E2 administration in the ArKO mouse leads to decreased PPARγ expression [33], which may cause the reduction in adiponectin we observed. Another possible mechanism for reducing adiponectin in the E2 treated ArKO was the observed increases in serum inflammatory markers TNFα, IL6 and MCP1, which are all known inhibitors of adiponectin.

Although E2 replacement in many cases of estrogen deficiency, such as menopause and ovariectomy, results in reduced adiposity and inflammation [34,35]; there are cases whereby estrogens can play a negative role in the development of inflammation. High or supra-physiological levels of estrogens as seen in third trimester pregnancy [36] are associated with increases in TNFα and leptin concentrations in addition to decreased adiponectin [37]. Riant and colleagues [38] also found increase in TNFα, IL6, MCP1 and F480 in ovariectomised mice on a high fat diet when treated with estrogens. Macrophage density was also increased in adipose tissue in this model which may account for the increased capacity of macrophages to produce cytokines [38]. Direct modulation of inflammation by estrogens is corroborated by the location of estrogen receptors (ERs) on cells of the immune system [39] and have been associated with modulating immune cell recruitment into estrogen sensitive tissue such as the uterus [40,41].

Subsequent increased MCP1 concentration observed in the female ArKO serum with E2 treatment and increased CD45 positive cells in the adipose tissue of female ArKO and ArKO+E2 mice suggest enhanced macrophage infiltration from the periphery which may explain the increases in serum and adipocytes TNFα and IL6 in these animals. However, the discrepancies between serum and adipose tissue MCP1 levels could be explained by the fact that MCP1 is not exclusively produced by adipose tissue [42]. Indeed, our data support the observation that estrogen actions on inflammation are still dependent on a myriad of factors such as target organ, concentration (biphasic effects) and the ER isoforms present [43]. Estrogens may be acting through ERα instead of ERβ to exert changes in adiposity since ERαKO mice of both sexes present increased adiposity but not ERβKO mice (for review see reference [15]); the former presents similar adiposity phenotypes to our estrogen-deficient ArKO mouse model [25].

Estrogen deficiency had neither inhibitory modifications in muscle or adipose Akt phosphorylation nor insulin stimulated *ex vivo* skeletal muscle glucose uptake. The high HDL levels [44,45] in female ArKO mice may counteract any reductions in Akt associated with insulin resistance in muscle and adipose tissues, leading to no significant changes in Akt phosphorylation in these ArKO tissues when compared to the WT.

## Conclusion

The ArKO mouse is an important estrogen responsive model which allows us to examine exogenous estrogen dosage on the relationship between hormones, adiposity and insulin resistance without the interference of uncontrolled endogenous estrogen production. Our observations provide an understanding into the complex mechanisms, including changes in Akt, TNFα, IL6 and MCP1 by which estrogen regulates obesity and insulin resistance. Estrogen administration in the ArKO mice leads to improvements in liver glucose homeostasis. However, simultaneous displays of increased inflammatory processes in the adipose tissue adipokines may be responsible for the lack of improved whole body glucose recovery after estrogen replacement.

## Supporting Information

S1 Table Body and gonadal adipose tissue weights of untreated ArKO (KO) versus placebo-treated ArKO mice. Body and adipose tissue weights in grams (g) six month-old female aromatase knockout untreated (KO, n = 10) compared to six-week placebo-treated KO (KOP, n = 4). Data are expressed as mean ± SD, no significant differences are detected.
(DOCX)

S1 Fig Glucose tolerance tests of 6 month-old untreated ArKO (KO) vs. placebo treated ArKO (KOP). Whole body glucose tolerance tests (GTT) were completed on fasted 6 month-old female untreated aromatase knockout (KO) and 6 weeks of placebo treated KO (KOP). (A) glucose tolerance test and (B) corresponding area under curve. Data are presented as mean ± SD, no significant differences are detected; sample sizes in brackets.
(PDF)
