# Supplementary material for: Effects of Estrogens on Adipokines and Glucose Homeostasis in Female Aromatase Knockout Mice
Source: PLoS One. 2015 Aug 28;10(8):e0136143. doi: 10.1371/journal.pone.0136143 (PMC4552801; doi:10.1371/journal.pone.0136143)
Supplement: S1 Table — Body and adipose tissue weights in grams (g) six month-old female aromatase knockout untreated (KO, n = 10) compared to six-week placebo-treated KO (KOP, n = 4). Data are expressed as mean ± SD, no significant differences are detected. (DOCX) [file pone.0136143.s001.docx]

**S1 Table. Body and gonadal adipose tissue weights of untreated ArKO (KO) versus placebo-treated ArKO mice**

Body and adipose tissue weights in grams (g) six month-old female aromatase knockout untreated (KO, n=10) compared to six-week placebo-treated KO (KOP, n=4). Data are expressed as mean ± SD, no significant differences are detected.

|  | **ArKO untreated** | **ArKO + Placebo** |
| --- | --- | --- |
| Number of mice | 10 | 4 |
| Body weight (g) | 32.9±6.68 | 31.75±9.36 |
| Gonadal adipose tissue (g) | 1.18±0.09 | 1.09±0.76 |
