# Supplementary material for: Effects of Estrogens on Adipokines and Glucose Homeostasis in Female Aromatase Knockout Mice
Source: PLoS One. 2015 Aug 28;10(8):e0136143. doi: 10.1371/journal.pone.0136143 (PMC4552801; doi:10.1371/journal.pone.0136143)
Supplement: S1 Fig — Whole body glucose tolerance tests (GTT) were completed on fasted 6 month-old female untreated aromatase knockout (KO) and 6 weeks of placebo treated KO (KOP). (A) glucose tolerance test and (B) corresponding area under curve. Data are presented as mean ± SD, no significant differences are detected; sample sizes in brackets. (PDF) [file pone.0136143.s002.pdf]

S1 Fig.

A)

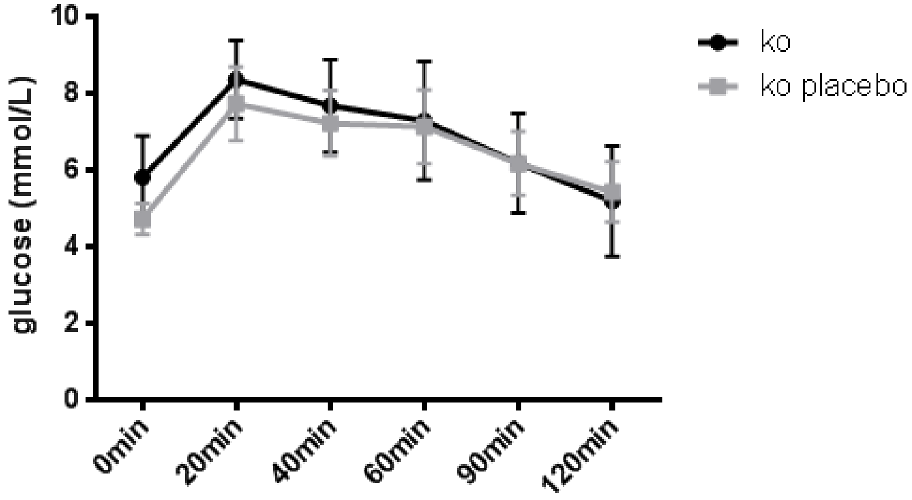

B)

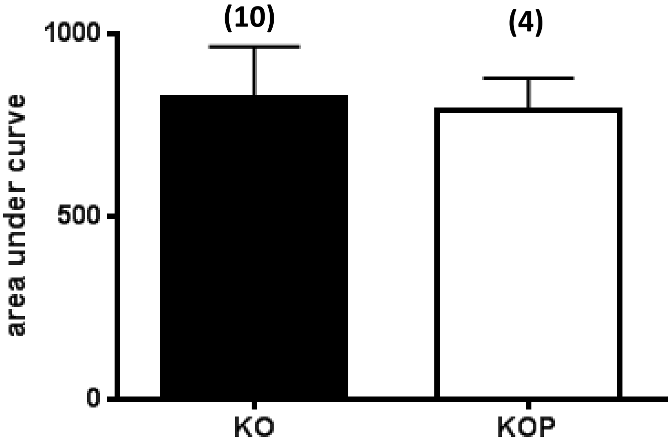

S1 Fig. Glucose tolerance tests of 6 month-old untreated ArKO (KO) vs. placebo treated ArKO (KOP). Whole body glucose tolerance tests (GTT) were completed on fasted 6 month-old female untreated aromatase knockout (KO) and 6 weeks of placebo treated KO (KOP) **(A)** glucose tolerance test and **(B)** corresponding area under curve. Data are presented as mean ± SD, no significant differences are detected; sample sizes in brackets.
